# Testing the potential of entomopathogenic nematodes in attract‐and‐kill and autodissemination approaches in the control of Queensland fruit fly, *Bactrocera tryoni*

**DOI:** 10.1002/ps.8416

**Published:** 2024-09-20

**Authors:** Sitaram Aryal, Geraldine Tilden, Markus Riegler

**Affiliations:** ^1^ Hawkesbury Institute for the Environment Western Sydney University Penrith Australia

**Keywords:** Tephritidae, yeast, attract‐and‐kill, dispersal, virulence, biocontrol

## Abstract

**BACKGROUND:**

Many studies have demonstrated that tephritid fruit fly larvae are highly susceptible to entomopathogenic nematodes (EPNs) and may become infected as they enter the soil to pupate. However, the susceptibility of adult tephritids and their suitability as EPN targets have been less studied. We performed laboratory assays with 12 Australian EPN strains of *Heterorhabditis bacteriophora*, *Heterorhabditis indica* and *Heterorhabditis zealandica* in adults of the Queensland fruit fly, *Bactrocera tryoni*. Infective juveniles were delivered in a yeast hydrolysate solution that is attractive to flies. We also measured the flight ability of adults up to 3 days after treatment.

**RESULT:**

Flies that consumed the EPN‐yeast preparation experienced 72.8–84% mortality. Between 33.5% and 46.2% of EPN‐treated adults were still able to fly before death following treatment, mostly within the first day, thereby contributing to EPN dispersal. Another 31.9–39.9% of EPN‐treated flies that were unable to fly died as a result of EPN treatment. Overall, >65% of flies that died following EPN treatment had visible signs of infection and EPN reproduction.

**CONCLUSION:**

Our study is foundational to the development of attract‐and‐kill and autodissemination approaches involving EPNs in fruit fly control. Furthermore, *H. indica* and *H. zealandica* strains showed the highest potential as biocontrol agents against adult flies. © 2024 The Author(s). *Pest Management Science* published by John Wiley & Sons Ltd on behalf of Society of Chemical Industry.

## Graphical abstract

Entomopathogenic nematodes (EPNs) delivered in a fly‐attractive aqueous yeast hydrolysate solution cause high levels of mortality in Queensland fruit fly. Infected flies can still fly and thereby disperse EPNs before the flies succumb to infection.

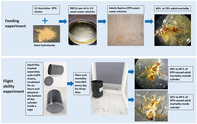

## 1 INTRODUCTION

The Queensland fruit fly, *Bactrocera tryoni* (Froggatt), Australia's most important horticultural pest species, causes significant damage in a wide range of crops, and has economic impacts in terms of production loss, management costs and market access restrictions.^1^, ^2^ It occurs throughout northern and eastern Australia, as well as on several South Pacific islands; it also experiences occasional outbreaks in other parts of Australia, New Zealand, other parts of the Pacific region and parts of the United States, where it is targeted by eradication measures.^3^, ^4^ Fruit fly management can include a combination of strategies^5^ such as orchard hygiene and field sanitation,^6^ protein bait sprays,^6^ cover sprays,^7^ male annihilation technique (MAT) relying on male lures,^8^, ^9^ other attract‐and‐kill strategies that include female attractants,^8^, ^10^ sterile insect technique (SIT)^11^, ^12^ and area‐wide management.^13^ Several of these strategies involve the use of synthetic insecticides^8^, ^14^, ^15^; however, some of these chemicals can have negative nontarget effects on pollinators and other beneficial insects, as well as humans and other animals, including residual direct and indirect effects on the soil and other elements of the environment.^8^, ^16^ Therefore, the use of many chemical insecticides has been restricted or banned.^7^ As a consequence, there is a need for alternative control measures, including biological control.^17^ A number of biological control agents such as parasitoids,^18^ entomopathogenic fungi^19^, ^20^, ^21^ and entomopathogenic nematodes (EPNs)^22^, ^23^, ^24^ have been tested against *B. tryoni*. EPNs form the active ingredient of biopesticides that are used widely against several pests around the world,^25^, ^26^, ^27^, ^28^, ^29^ and they can be an important component in integrated pest management.^30^, ^31^ Therefore, the application of EPNs can be an alternative control measure with advantages over the use of synthetic chemicals, as EPNs are safe to humans, other animals and the environment.^32^

The EPNs of the families Steinernematidae and Heterorhabditidae are small soil‐borne invertebrates that are associated with symbiotic bacteria of the genera *Xenorhabdus* and *Photorhabdus*, respectively. The infective juvenile (IJ) nematodes enter their hosts via natural openings or through the soft intersegmental regions.^33^ Once inside the hemocoel, the IJs release bacteria that multiply and kill the insect host within 48 h. The nematodes can then complete two to three generations within the insect host, after which the free‐living IJs emerge from the corpse to seek new host individuals.^33^ EPNs have been successfully used as biological control agents against many pest insects with developmental stages in the soil,^34^, ^35^, ^36^ including tephritid flies.^37^ The damaging stages of tephritid pests such as *B. tryoni* are aboveground, yet their fully developed 3rd‐instar larvae leave infested fruit, drop to the ground and burrow into the soil where they pupate.^38^ Therefore, fully developed larvae and pupae of tephritids can be targeted by EPN soil applications.^39^, ^40^, ^41^ EPN soil applications may also work against other non‐tephritid pest fruit flies such as *Drosophila suzukii* (Matsumura).^42^ Several laboratory studies have been conducted to test the effectiveness of different species of EPNs against tephritid larvae^22^, ^23^, ^24^, ^43^ and pupae.^22^, ^23^, ^44^, ^45^ Besides the larval and pupal stages, adult tephritids also spend a short time in the soil when emerging from the pupae and crawling to the soil surface. Therefore, adult flies can also be targeted by soil applications of EPNs. However, there are relatively few studies that have tested the EPN susceptibility of adult tephritids.^46^, ^47^, ^48^ Besides affecting the overall fruit fly population, targeting adult tephritids could also help in EPN dispersal and persistence in fruit and vegetable production. The flight of EPN‐treated adults may contribute to the dissemination of EPNs before these adults succumb to the infection, including in locations frequented by other individuals of the target pest. This could be where adult flies rest and forage, such as on fallen fruit on the ground or in the tree canopy, as well as at mating aggregation sites in the tree canopy.^49^ At these locations, EPN‐infected fruit fly cadavers may facilitate the propagation of EPNs and the released IJs may infect other fruit fly individuals visiting these locations. Therefore, the dissemination of EPNs by infected flies may aid in effective fly control.^50^ Such strategies that involve the dissemination of entomopathogens by the target species are known as autodissemination and have been tested for several insect species.^51^, ^52^, ^53^ However, the development of autodissemination strategies of entomopathogens, including against tephritid pests, has so far focused mostly on entomopathogenic fungi.^37^ Besides this, only very few studies have investigated the potential of autodissemination strategies for EPNs against pest insects, such as the red fire ant *Solenopsis invicta* Buren,^54^ the goat moth *Cossus cossus* Linnaeus, the lawn caterpillar *Spodoptera cilium* Guenée,^55^ the chive gnat *Bradysia odoriphaga* Yang and Zhang,^56^ and the pine weevil *Hylobius abietis* (Linnaeus).^57^

Furthermore, adult tephritids may be difficult to control by conventional EPN applications such as soil treatments. However, fruit flies are attracted to a variety of volatiles and resources, such as fruit odors and plant‐derived attractants (parapheromones), as well as protein sources such as yeast hydrolysate.^58^, ^59^, ^60^ Some parapheromones are only attractive to males,^61^ whereas protein sources are attractive to both females and males.^62^ Adult female and male tephritid fruit flies including those of the genus *Bactrocera* are attracted to yeast,^62^ and yeast as a protein source is an important factor in fruit fly physiology and development, such as in oogenesis,^63^, ^64^, ^65^ male sexual development,^66^ mating behavior^67^ and development of attraction to cue lure.^60^

Attractants could also be used to attract fruit flies to locations or feeding stations where they become infected by EPNs, but this has not yet been tested. Such attract‐and‐kill strategies have been developed for the control of fruit fly and include the use of male lures (in MAT) and protein sources as bait together with insecticides as the killing agent.^61^ More recently, research also tested the use of entomopathogenic fungi as biopesticides in attract‐and‐kill strategies.^68^, ^69^ Likewise, EPNs may be used as an alternative to chemical insecticides in attract‐and‐kill systems but, to the best of our knowledge, this has so far not been tested for EPNs. A similar approach has been developed and implemented for the control of the wood wasp *Sirex noctilio* Fabricius by attracting wood wasps to bait trees containing the parasitic nematode *Deladenus siridicola*.^70^

Before the potential use of EPNs in attract‐and‐kill and autodissemination strategies against tephritid pests such as *B. tryoni* can be examined in field settings, it is essential to perform studies in controlled laboratory environments. Therefore, the aim of our study was to investigate the susceptibility of *B. tryoni* adults to 12 Australian‐native EPN strains isolated from Australian soils^71^ that had previously been tested against immature stages of *B. tryoni*.^23^ Then we tested whether we can combine these 12 EPN strains with a yeast hydrolysate solution attractive to both female and male adults, to bring about EPN‐induced fly mortality. We further investigated for four of these EPN strains whether treated adults were able to fly after becoming infected, thereby potentially contributing to the dispersal of EPNs.

## 2 MATERIALS AND METHODS

### 2.1 EPN collection and storage

Our study used 12 EPN strains of *Heterorhabditis bacteriophora*, *Heterorhabditis indica* and *Heterorhabditis zealandica,* isolated from across eastern Australia in 2018 and 2019 (Table 1), and characterized in a previous study.^71^ For this previous study, 198 soil samples were collected from 71 sites in Queensland, New South Wales and Victoria following a random sampling method described previously by Orozco *et al*.^72^ From each site a composite 3 kg topsoil sample was collected at a depth of 0–30 cm. The soil was placed in ziplock plastic bags, stored at 15 °C and baited for EPNs within 5 days of sampling using mealworm (*Tenebrio molitor* Linnaeus) and *B. tryoni* larvae.^71^ After isolation and characterization, the 36 new EPN strains were maintained at 15 °C by regular infection of mealworm larvae and harvest of IJs for new infections. For the experiments in this study, we selected 12 EPN strains that had previously performed well in laboratory assays of 32 EPN strains against *B. tryoni* larvae and pupae.^22^, ^23^ We harvested fresh IJs of the 12 EPN strains after infecting mealworm larvae independently for each experimental replicate. The infected cadavers were then individually transferred to White traps^73^ modified as per Kaya and Stock.^74^ After 2 weeks, IJs were harvested from the White traps and stored in Ringer's solution [9.0 g salt (NaCl), 0.42 g potassium chloride (KCl), 0.37 g calcium chloride dehydrate (CaCl_2_.2H_2_O) and 0.2 g sodium bicarbonate (NaHCO_3_) dissolved in 1 L distilled water] at 15 °C and used within a week. The motility of each IJ batch was observed under the microscope and only IJ batches with <2% mortality were used in the experiments.

**Table 1:** Entomopathogenic nematode (EPN) strains used in this study were previously collected from New South Wales and Queensland in Australia in 2018/2019, identified and characterized,^71^ and tested against larval and pupal stages of *Bactrocera tryoni* ^22^, ^23^

EPN strains	EPN species	Bacterial symbiont	Discoloration of EPN‐infected larvae	Source of isolation
Hb.HIE1	*Heterorhabditis bacteriophora*	*Photorhabdus laumondii*	Brick red	Richmond, NSW
Hb.HIE2	*H. bacteriophora*	*P. tasmaniensis*	Brick red	Richmond, NSW
Hi.ECCH	*H. indica*	n.d.	Brick red	Richmond, NSW
Hi.HRN2	*H. indica*	n.d.	Brick red	Heron Island, QLD
Hi.LMBT	*H. indica*	*P. laumondii*	Brick red	Lady Musgrave Island, QLD
Hi.QF6	*H. indica*	*P. tasmaniensis*	Brick red	Palmwoods, QLD
Hi.QGLB	*H. indica*	n.d.	Brick red	Duingal, QLD
Hz.BB1	*H. zealandica*	n.d.	Green	Batemans Bay, NSW
Hz.BB3	*H. zealandica*	n.d.	Green	Batemans Bay, NSW
Hz.NAR1	*H. zealandica*	n.d.	Green	Narara, NSW
Hz.NAR2	*H. zealandica*	*P. namnaonensis*	Brick red	Narara, NSW
Hz.NAR3	*H. zealandica*	n.d.	Green	Narara, NSW

All isolates were baited from soil with *Tenebrio molitor* larvae except Hi.LMBT which was baited with *B. tryoni* larvae.

### 2.2 Fruit fly culture

We used a laboratory population of *B. tryoni* which was established from flies collected on the Hawkesbury campus of Western Sydney University in Richmond, New South Wales, in 2009.^75^ Since then, this fly population had been maintained (six to eight generations per year; ≈80 generations) in a glasshouse chamber at 25 °C and 70% relative humidity (RH).^24^ Eggs were collected from adult flies kept in cages (30 × 30 × 30 cm) using 120‐mL cups filled with larval diet.^64^ The cups were covered with parafilm perforated with needles and provided to the caged fruit flies to allow oviposition into the larval diet for 2 hours. The cups were then transferred to a container which had a fine layer of sterile sand sprinkled over the base for pupation and a mesh screen installed in the lid. The pupae were collected and placed into cages for adults to emerge. Adults were provided with water, sugar and yeast hydrolysate.^64^

### 2.3 Adult feeding and infection experiment

The aim of the adult feeding and infection experiment was to test whether adult fruit flies could become EPN‐infected by providing adults with an aqueous yeast hydrolysate solution containing IJs. For this, we tested each of the 12 EPN strains against adult *B. tryoni* in separate cages (30 × 30 × 30 cm) and included one control cage without EPNs. Each of the 13 cages was supplied with 70 *B. tryoni* pupae, as well as water and sugar for emerging flies. Once >80% of the flies had emerged, the 12 EPN treatment cages were provided with 20 000 IJs (one EPN strain per cage) in 40 mL yeast hydrolysate (1% w/v) solution (500 IJs mL^−1^) dispensed equally into two 9‐cm Petri dishes placed on the bottom of the cage without any other food supply (Supporting information Fig. S1). This concentration of 500 IJs mL^−1^ was chosen based on previous virulence experiments with these 12 and other EPN strains in *B. tryoni* larvae and pupae where this concentration caused high mortality.^23^ The flies in the control cage received 40 mL yeast hydrolysate (1% w/v) solution without IJs, also dispensed into two Petri dishes placed on the bottom of the cage without any other food supply. The experiment was performed at room conditions of 21.5 ± 2.5 °C, 72 ± 7% RH and natural light. Mortality of the emerged flies was recorded after 7 days. All dead adults were incubated for a further 3 days [total of 10 days after treatment (DAT)] to allow EPN development and then dissected to confirm the presence of EPNs as a visible sign of infection. The experiment included 12 EPN treatment cages (one per strain) and one control cage. The experiment with the 13 cages was replicated on five occasions over time (total of 65 cages).

### 2.4 Adult infection and flight ability experiment

The aim of the adult infection and flight ability experiment was to assess the flight ability of EPN‐treated adult flies at 1, 2 and 3 DAT with EPNs, and, hence, the capability of infected flies to disperse EPNs. For this we developed a flight ability test based on the product quality control guidelines for mass‐reared flies used in SIT programs.^76^ For this experiment we selected four of the 12 EPN strains, one each for *H. bacteriophora* (Hb.HIE1) and *H. indica* (Hi.LMBt), and two for *H. zealandica* (Hz.BB1 and Hz.NAR2, each with another bacterial associate causing different coloration of larval cadavers) (Table 1).^22^, ^23^ Hi.LMBt was included because it performed well in the adult feeding experiment and had originally been baited from soil using *B. tryoni* (Table 1). The other three strains were selected based on their performance in the adult feeding experiment. The experiment was performed, together with a control treatment, in a glasshouse chamber at 25 °C, 70% RH and natural light conditions. Furthermore, for each of the four EPN treatments and the control, three different cages (total of 15 cages) were set up to test EPN infection and flight ability 1, 2 and 3 DAT. For this, three cohorts of 70 *B. tryoni* adults (2–3 days old) were chilled for a short time (4 °C for 15 min) to reduce their activity for handling and treatment. Each of the three cohorts were then placed on a filter paper in a 9‐cm Petri dish and carefully covered with the Petri dish lid which contained another filter paper. The two filter papers had each been treated with 5000 IJs in 1 mL Ringer's solution delivered with a pipette. Control flies received two filter papers, each treated with 1 mL Ringer's solution without IJs. Afterwards, the flies were incubated between the filter papers at 25 °C for 6 h. Each cohort of adults was then placed on a 14‐cm Petri dish (with a black base) holding a black cylindrical PVC tube (10 cm long × 14 cm diameter) internally covered with fluon to prevent flies from crawling out (Fig. S2). Each black Petri dish carrying the adults and a black cylinder was placed into a cage and kept without food at 25 °C, 70% RH and natural light conditions. The number of live and dead adult flies inside (nonfliers) and outside (fliers) the black cylinder was counted at 1 DAT (cage 1), 2 DAT (cage 2) and 3 DAT (cage 3) with EPNs. Dead flies were incubated for a further 7 days (total of 7–10 DAT) before dissection to assess EPN infection. The experiment included three cages (1, 2 and 3 DAT) for each of the four EPN strains, as well as three control cages (1, 2 and 3 DAT). The experiment with the 15 cages was replicated on five occasions over time (total of 75 cages).

### 2.5 Statistical analysis

The mortality data were not normally distributed and were arcsine‐transformed before passing the Shapiro–Wilk test. The data were then subjected to one‐way ANOVA (adult feeding experiment) and two‐way ANOVA (flight ability experiment). Tukey's honestly significant difference (HSD) tests were conducted for multiple comparisons. All data analyses were performed in R v4.2.1.^77^ Furthermore, the overall mortality after EPN treatments was corrected for the mortality seen in the control treatment using Abbott's formula^78^:

$$\begin{array}{l} \text{Corrected}\;\mathrm{EPN}-\text{caused mortality}=\left[\left(\%\text{test mortality}–\%\text{control mortality}\right)/\left(100–\%\text{control mortality}\right)\right]\times 100 \end{array}$$

This corrected EPN‐caused mortality is cumulative as it accounted for two categories of flies: dead EPN‐treated flies with visible EPN infection upon dissection (resulting in the reproduction of EPNs) and dead EPN‐treated flies without visible EPN infection upon dissection (not resulting in the reproduction of EPNs).

## 3 RESULTS

### 3.1 Adult feeding experiment

**Figure 1:**
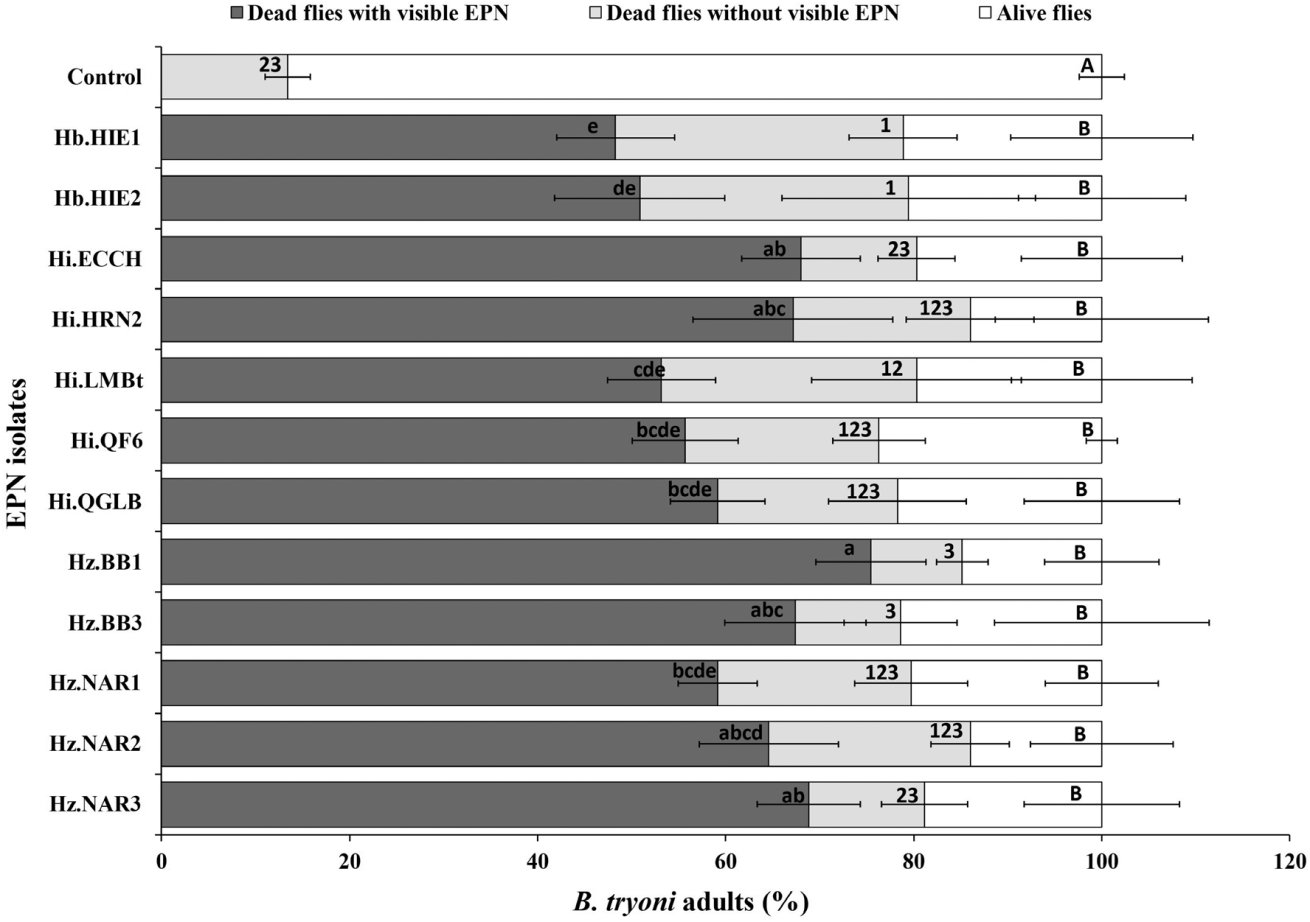
Percentage of dead and live *Bactrocera tryoni* adults after 7 days of feeding on an aqueous yeast hydrolysate (1% w/v) solution containing entomopathogenic nematodes (EPNs) (500 IJs mL^−1^). The dark and light gray sections represent dead flies with and without visible EPNs when dissected after three additional days of incubation, and the white sections represent live flies. Error bars indicate the standard deviation across five replicates. Different letters and numbers next to the error bars indicate that means are significantly different (lowercase letters for comparison of dead flies with visible EPNs across treatments; numbers for comparison of dead flies without visible EPNs across treatments; uppercase letters for comparison of live flies across treatments) (Tukey's honestly significant difference test, *P* < 0.05).

**Figure 2:**
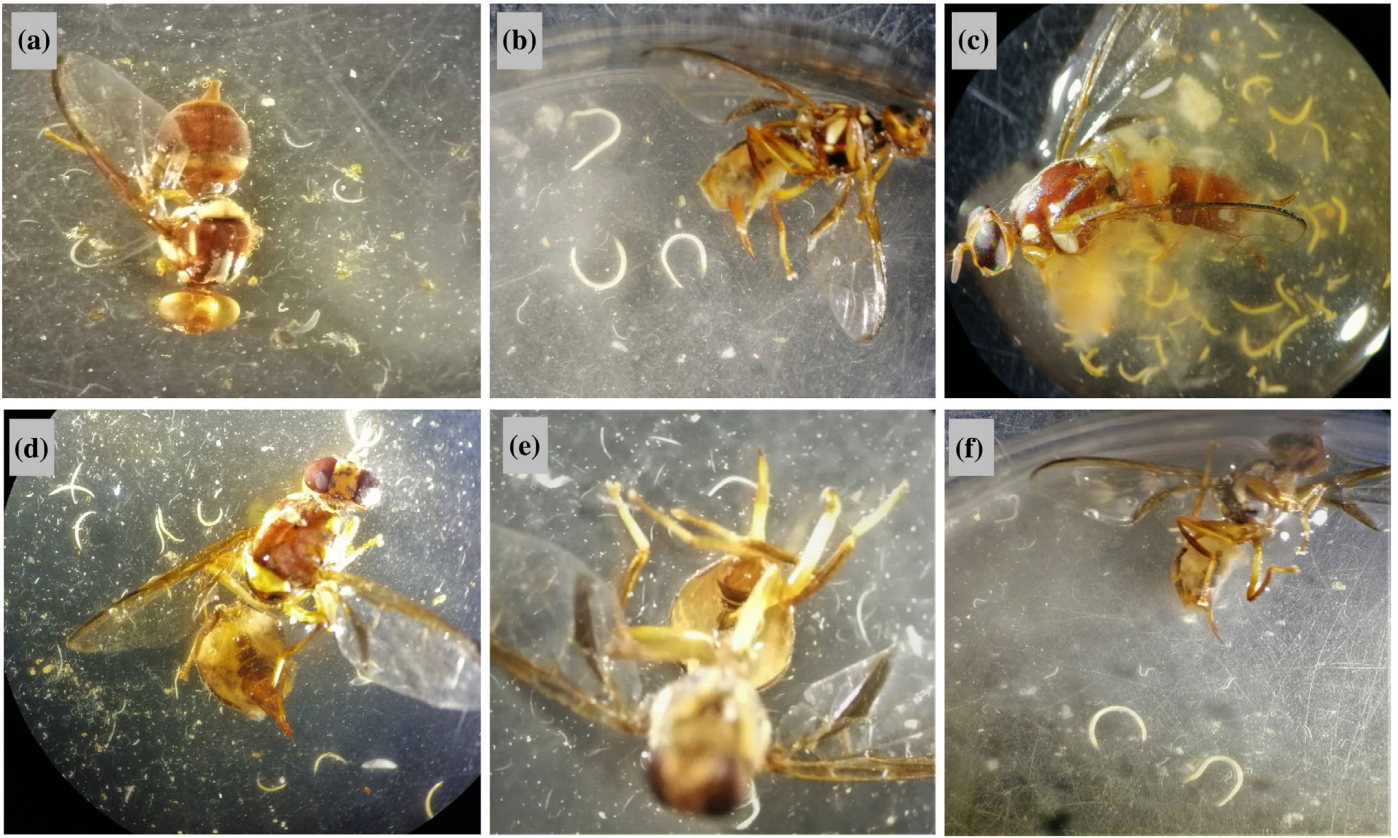
*Bactrocera tryoni* adults infected with (a) *Heterorhabditis bacteriophora* Hb.HIE1, (b) *Heterorhabditis indica* Hi.ECCH, (c) *Heterorhabditis indica* Hi.LMBt, (d) *Heterorhabditis indica* Hi.QF6, (e) *Heterorhabditis zealandica* Hz.BB1 and (f) *Heterorhabditis zealandica* Hz.NAR2. Note that dead flies were not discolored owing to EPN infection; by contrast such discoloration is seen in EPN‐infected larvae.^22^, ^23^

Adults of *B. tryoni* provided with 500 IJs mL^−1^ in yeast hydrolysate solution experienced significant mortality with all 12 EPN strains (Fig. 1). After the incubation period EPNs were found to be present in many dead flies indicating that EPNs were able to reproduce in adult cadavers (Fig. 2). Some dead flies, however, had no signs of EPN presence. Therefore, the percentage of dead flies was categorized as dead flies with and without visible EPNs (Fig. 1). The detection of EPNs ranged from 48.3 ± 6.3% of treated individuals for *H. bacteriophpora* Hb.HIE1 to 75.4 ± 5.8% of treated individuals for *H. zealandica* Hz.BB1 (Fig. 1). The overall mortality observed after EPN application, irrespective of the detection of EPN infections, differed significantly among EPN strains (*F*_12,52_ = 17.2, *P* < 0.0001) and ranged between 76.2 ± 1.6% for *H. indica* Hi.QF6 and 85.9 ± 11.3% for *H. indica* Hi.HRN2 (Fig. 1). However, after correction of the overall mortality of EPN‐treated flies with the mortality seen in control flies, the EPN‐caused mortality was not different between EPN strains (*F*_11,48_ = 0.71, *P* = 0.72) and ranged from 72.8 ± 3.4 for *H. indica* Hi.QF6 to 84.0 ± 12.9% for *H. indica* Hi.HRN2 (Fig. S3).

### 3.2 Flight ability experiment

**Figure 3:**
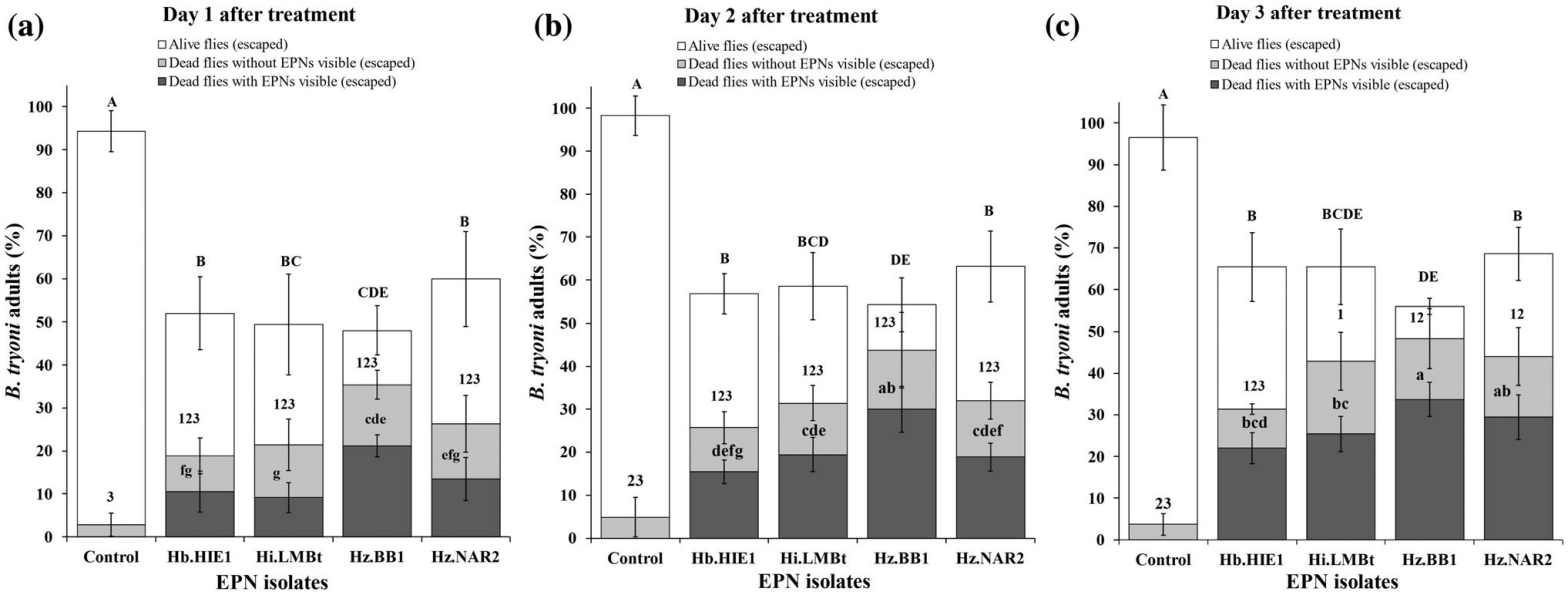
Percentage of dead and live *Bactrocera tryoni* adults that were able to fly out of the test cylinder (escaped fliers), as scored at (a) 1 day after treatment (DAT) (cage 1), (b) 2 DAT (cage 2), and (c) 3 DAT (cage 3) with EPNs. The dark and light gray sections represent dead flies with and without visible EPNs when dissected after 7 days of additional incubation, and the white sections represent live flies. Error bars indicate the standard deviation across five replicates. Different letters and numbers on top of the error bars indicate that means are significantly different from each other among all 3 time periods (lowercase letters for comparison of dead flies with visible EPNs across treatments; numbers for comparison of dead flies without visible EPNs across treatments; uppercase letters for comparison of live flies across treatments) (Tukey's honestly significant difference test, *P* < 0.05).

**Figure 4:**
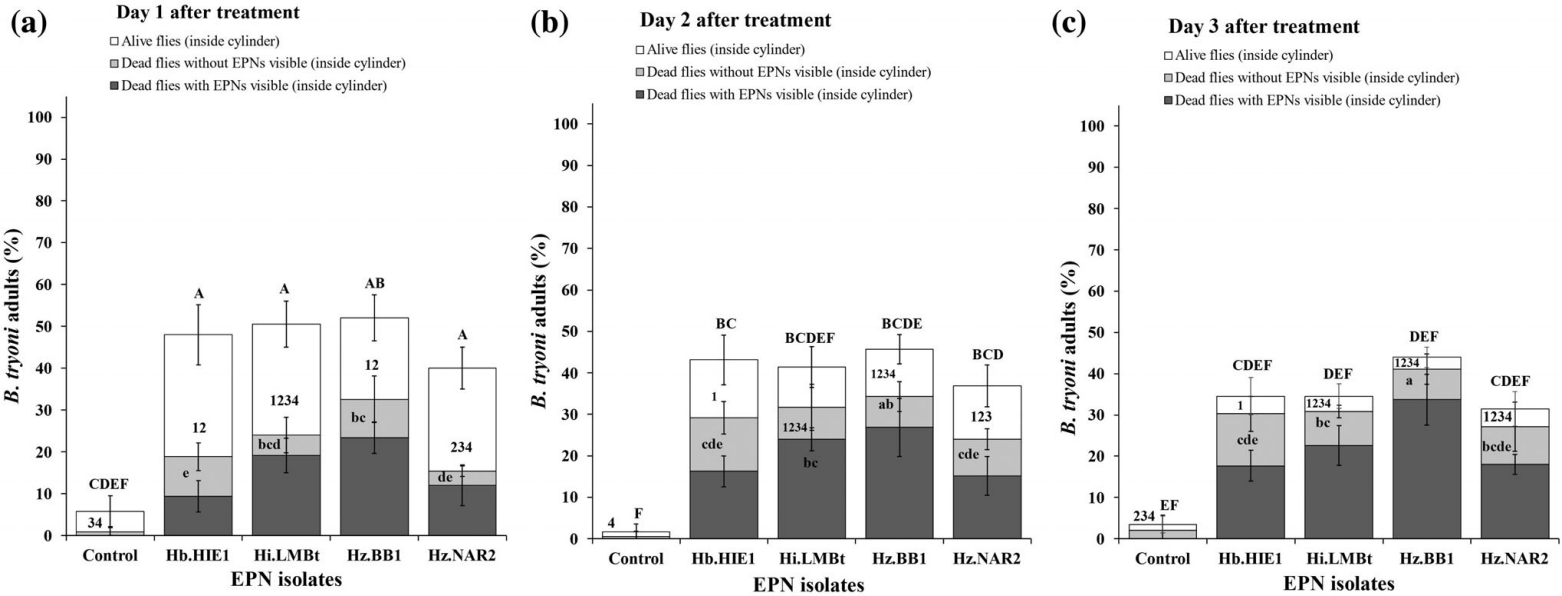
Percentage of dead and live *Bactrocera tryoni* adults that were unable to fly out of the flight ability test cylinder (inside cylinder, nonfliers), as scored at (a) 1 day after treatment (DAT) (cage 1), (b) 2 DAT (cage 2) (c) 3 DAT (cage 3) with EPNs. The dark and light gray sections represent dead flies with and without visible EPNs when dissected after 7 days of additional incubation, and white sections represent live flies. Error bars indicate the standard deviation across five replicates. Different letters and numbers on top of the error bars indicate that means are significantly different from each other among all 3 time periods (lowercase letters for comparison of dead flies with visible EPNs across treatments; numbers for comparison of dead flies without visible EPNs across treatments; uppercase letters for comparison of live flies across treatments) (Tukey's honestly significant difference test, *P* < 0.05).

Similar to the adult feeding experiment, all four EPN strains were able to infect and cause mortality in *B. tryoni* adults after they had been in contact with the EPN‐treated filter papers. After 1 [Fig. 3(a)], 2 [Fig. 3(b)] and 3 DAT [Fig. 3(c)], 54.2 ± 12.3%, 60.0 ± 11.2% and 65.3 ± 10.6% of the EPN‐treated flies (averaged across the four EPN strains) were able to fly out from the flight ability test cylinder (fliers) compared with 94.2 ± 4.7%, 98.2 ± 2.3% and 96.6 ± 5.6% of the control flies, respectively. By contrast, at 1 [Fig. 4(a)], 2 [Fig. 4(b)] and 3 DAT [Fig. 4(c)], 45.7 ± 12.3%, 40.0 ± 11.2% and 34.6 ± 10.6% of the EPN‐treated flies (averaged across five replicates) were unable to fly out of the cylinder (nonfliers) compared with 5.7 ± 4.7%, 1.7 ± 2.3% and 3.4 ± 5.6% of the control flies, respectively.

**Figure 5:**
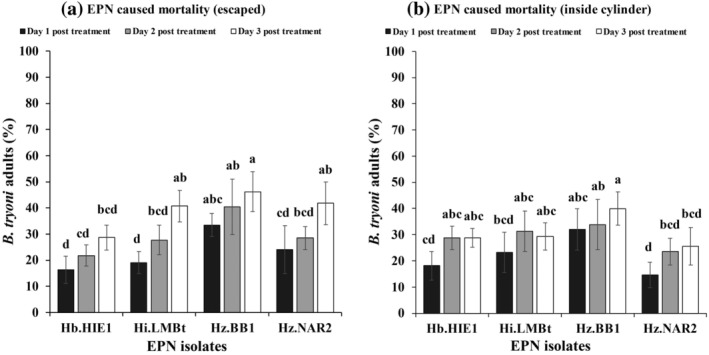
Corrected percentage of mortality of *Bactrocera tryoni* adults at 1 (dark gray), 2 (light gray) and 3 days (white) after treatment with EPN, corrected by the mortality observed in the control treatment: (a) fliers that were able to fly out of the flight ability test cylinder (escaped) and (b) nonfliers that were unable to fly out of the cylinder (inside the cylinder). Error bars indicate the standard deviation across five replicates. Different letters next to the error bars indicate that means are significantly different (Tukey's honestly significant difference test, *P* < 0.05).

After correction for the mortality observed in the control, the EPN‐caused mortality of fliers (outside the cylinder) was 33.5 ± 4.4%, 40.4 ± 10.6% and 46.2 ± 7.7% at 1, 2 and 3 DAT, respectively [Fig. 5(a)]. By contrast, the corrected EPN‐caused mortality of nonfliers inside the cylinder was 31.9 ± 7.9%, 33.9 ± 9.6% and 39.9 ± 6.3% at 1, 2 and 3 DAT with EPNs [Fig. 5(b)]. In comparison, corrected EPN‐caused mortality of 21.0 ± 2.6% to 34.0 ± 4.1% of EPN‐treated fliers were visibly EPN‐infected when dissected after 7 days of additional incubation [Fig. 3(a)–(c)]. By contrast, 23.0 ± 3.7% to 34.0 ± 6.1% of EPN‐treated nonfliers were visibly EPN‐infected when dissected after 7 days of additional incubation [Fig. 4(a)–(c)]. The percentage of visibly EPN‐infected fliers differed significantly with the number of days after EPN treatment (*F*_2,57_ = 14.8, *P* < 0.001) and among EPN strains (*F*_3,56_ = 8.81, *P* < 0.001), with a significant interaction between number of days and EPN strains (*F*_11,48_ = 9.55, *P* < 0.001). There were significant differences in the percentage of EPN‐infected fliers within 1 (*F*_3,16_ = 8.61, *P* = 0.0012), 2 (*F*_3,16_ = 12.67, *P* = 0.0001) and 3 DAT (*F*_3,16_ = 6.58, *P* = 0.0041) with EPNs. We further found significant differences in the percentage of visibly EPN‐infected fliers between the time periods of 1 and 2 DAT (*F*_3,36_ = 10.52, *P* < 0.001), 1 and 3 DAT (*F*_3,36_ = 3.54, *P* = 0.02), and 2 and 3 DAT (*F*_3,36_ = 11.64, *P* < 0.001) with EPNs. After correction of the overall mortality of EPN‐treated flies with the control mortality, the EPN‐caused mortality of fliers differed significantly between the three time periods and the four EPN strains (*F*_11,48_ = 11.15, *P* < 0.001) (Table S1).

## 4 DISCUSSION

We found that *B. tryoni* adults can be attracted to yeast hydrolysate solution containing EPNs, become EPN‐infected and experience substantial EPN‐caused mortality. Furthermore, we found that EPN‐infected flies can fly and disperse EPNs up to 3 DAT with EPNs. Therefore, our research shows that there is potential for the development of EPNs in attract‐and‐kill and autodissemination approaches against pest fruit flies.

We are not aware of any previous reports that have tested adult fruit fly susceptibility to EPNs by providing them to flies within an attractive food source. A similar approach, however, has been used in the biological control of the wood wasp *S. noctilio* by inocculating bait trees with the parasitic nematode *D. siridicola*.^70^ Our study showed that the application of EPNs in an aqueous yeast hydrolysate solution helped to attract adult *B. tryoni* to EPNs resulting in EPN‐caused mortality in flies that had not been treated directly. In this way, EPNs were able to infect and reproduce in up to 75% of adult flies. This corroborates the recent findings by Aryal *et al*.^23^ who recorded a high reproductive potential of EPNs in *B. tryoni* larvae and pupae. Furthermore, previous sand plate experiments without any attractant recorded adult mortality of up to 60% in *Ceratitis rosa* Karsch and *C. capitata*,^79^ up to 75% in *Dacus ciliatus* Loew^46^ and up to 60% in *Bactrocera zonata* (Saunders).^80^ Besides the different experimental setups, the EPN treatment concentrations used in these assays were substantially higher (200 IJs/50 μL; 200 IJs cm^−2^) than in our study that achieved a similar mortality with EPNs provided at a lower concentration (500 IJs mL^−1^).

Furthermore, we found that EPNs did not reproduce in up to 17% of treated adult flies that died following EPN treatment. This could possibly be a consequence of other microbes or contaminating factors being present in these EPN‐infected flies leading to poor development, intoxication and death of EPNs inside the cadaver. Upadhyay and Mohan^81^ found that the presence of nonsymbiotic bacteria such as *Bacillus subtilis* suppresses EPN development in the wax moth *Galleria mellonella* (Linnaeus) killed by EPNs.

Diverse field environments are not always favorable for EPN survival and persistence which are largely affected by soil temperature,^22^ soil moisture,^82^ soil type^25^ and host availability,^83^ as well as the foraging strategy and dispersal capability of the EPN strains in the soil.^84^ Although EPN‐infected flies can support EPN reproduction and therefore contribute to EPN persistence in the application area, infected flies could also be an agent for EPN dispersal. Our experiment showed that the largest percentage of flies (up to 60%) left the flight ability cylinder within 1 DAT with EPNs. The percentage of fliers observed across the 2‐day period (up to 63%) and 3‐day period (up to 69%) were only minimally higher than that from the 1‐day period. Furthermore, up to 34% of adults treated with EPNs had visible EPN infections and were still able to fly. Therefore, flies could act as a dispersal agent from the time of infection until they succumb to death. A similar percentage of treated flies showed visible EPN infections and were unable to fly, and these infected flies would contribute to the further establishment of EPN populations in the application area. We further note that, because of our experimental protocols in which we kept treated flies immobilized in Petri dishes for 6 h before the flight ability test, our values may underestimate the percentage of flies that could disperse EPNs. It has been found previously that in the field, the majority of *B. tryoni* individuals do not disperse very far (<500 m).^85^, ^86^ Hence, it can be expected that most infected flies would remain in the vicinity of the application area and disperse EPNs to locations frequented by other individuals (e.g. where they rest, forage and aggregate for mating). Any IJs released from infected fly cadavers could then contribute to new infections in other fly individuals and the persistence of EPNs in the environment.

The results of our experiment on the flight ability of EPN‐treated flies corroborated the findings of Garriga *et al*.^50^ who found that 21.4% of *D. suzukii* infected with EPNs could fly for 60 h after EPN treatment resulting in EPN dispersal. Somewhat related to the autodissemination approach is the release of EPN‐infected insects rather than the application of EPN suspensions. For example, previous research found that the application of EPN‐infected root weevil *Diaprepes abbreviatus* (Linnaeus)^87^ and *G. mellonella* ^54^ cadavers caused higher insect mortality in targeted pest populations than the application of EPNs in aqueous solution. Therefore, the dispersal of EPNs by infected fruit flies may not only help the dissemination of EPNs into new locations, but also may increase EPN‐induced mortality and aid in the wider control of fruit fly by these pathogens.

## 5 CONCLUSIONS

We found that it is possible to deliver EPNs via an aqueous yeast hydrolysate solution to attract adult *B. tryoni* and cause fly mortality. Considering that the use of EPNs in biological control of pest insects, including fruit flies, focusses on the application of IJs to the soil,^39^, ^40^, ^41^ the delivery of EPNs directly to adult flies via an adult attractant is a novel way to target these pests with EPNs outside soil environments. Although *B. tryoni* pupates in the soil from where adults then emerge, most of the adult stage of *B. tryoni* and other tephritids remains above ground. The application of EPNs in a yeast solution, for example within a feeding station, may expose adults to EPNs that then cause infection in adults. Infected flies could then help increase the establishment of EPNs in the application area and nearby, thereby contributing to wider fruit fly control. Given that our studies were conducted in laboratory and glasshouse environments, future studies should investigate attract‐and‐kill and autodissemination strategies using EPNs in semi‐field and field cage settings. Furthermore, more research needs to be done to further develop EPN applications and formulations for attract‐and‐kill and autodissemination strategies in fruit fly control, and in biological control of pest insects more widely.

## AUTHOR CONTRIBUTIONS

SA and MR conceptualized and designed the experiments. SA performed the experiments with the technical support of GT. SA analyzed the data. SA and MR wrote the manuscript with input from GT. MR was responsible for research funding.

## CONFLICT OF INTEREST

The authors declare no conflict of interest.

## Supporting information


**Data S1:** Supporting information.

## Data Availability

Research data are not shared.
