# Supplementary material for: Testing the potential of entomopathogenic nematodes in attract‐and‐kill and autodissemination approaches in the control of Queensland fruit fly, Bactrocera tryoni
Source: Pest Manag Sci. 2024 Sep 20;81(1):160–9. doi: 10.1002/ps.8416 (PMC11632213; doi:10.1002/ps.8416)
Supplement: Supplementary file 1 — Data S1: Supporting information. [file PS-81-160-s001.docx]

**SUPPLEMENTARY INFORMATION**

**Supplementary Table 1.** Statistic test results for visibly EPN-infected *Bactrocera tryoni* adults that flew out (fliers) and did not fly out (non-fliers) of the flight ability test cylinder after EPN application. The experiment was conducted with four EPN strains over one to three days.

|  | **Condition** | **Factor** | **F-statistic** | **DF** | ***p* value** | **Remarks** |
| --- | --- | --- | --- | --- | --- | --- |
| Dead flies with visible EPNs | Fliers that escaped  cylinder | 1 day | 8.61 | 3, 16 | 0.0012 | - |
|  |  | 2 days | 12.67 | 3, 16 | 0.0001 | - |
|  |  | 3 days | 6.58 | 3, 16 | 0.0041 | - |
|  |  | 1 day & 2 days | 10.52 | 3,36 | <0.0001 | - |
|  |  | 1 day & 3 days | 3.54 | 3,36 | <0.0001 | - |
|  |  | 2 days & 3 days | 10.27 | 3,36 | <0.0001 | - |
|  |  | Days: EPN strains | 17.87 | 11,48 | <0.0001 | - |
|  |  | Days: EPN strains | 11.15 | 11, 48 | <0.0001 | Corrected mortality with control |
|  | Non-fliers that remained inside  cylinder | 1 day | 12.04 | 3, 16 | 0.0002 | - |
|  |  | 2 days | 7.11 | 3, 16 | 0.0029 | - |
|  |  | 3 days | 14.03 | 3, 16 | <0.0001 | - |
|  |  | 1 day & 2 days | 14.98 | 3,36 | <0.0001 | - |
|  |  | 1 day & 3 days | 14.56 | 3,36 | <0.0001 | - |
|  |  | 2 days & 3 days | 17.83 | 3,36 | <0.0001 | - |
|  |  | Days: EPN strains | 11.13 | 11,48 | <0.0001 | - |
|  |  | Days: EPN strains | 5.69 | 11, 48 | <0.0001 | Corrected mortality with control |

**SUPPLEMENTARY FIGURES**


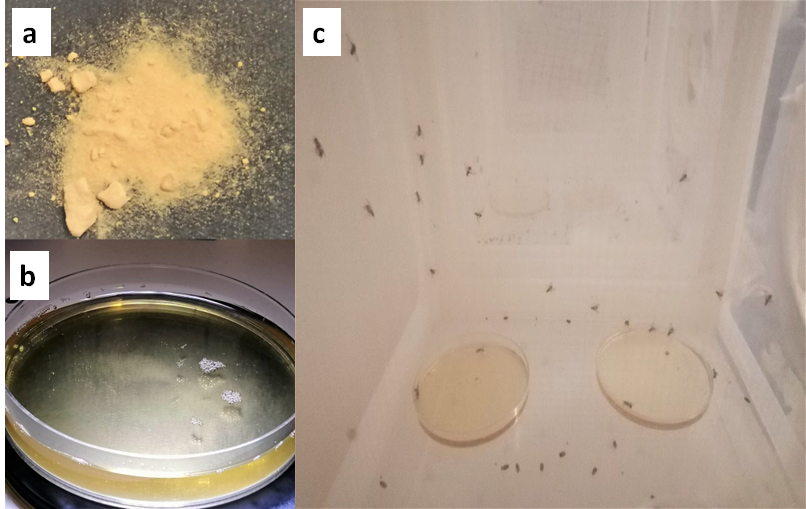


**Supplementary Fig. 1. a)** Yeast hydrolysate used as fruit fly attractant; **b)** aqueous EPN-yeast hydrolysate (1% w/v) solution containing 500 IJs/mL (control is without IJs); **c)** cage with aqueous EPN-yeast hydrolysate solution and *Bactrocera tryoni* adults.


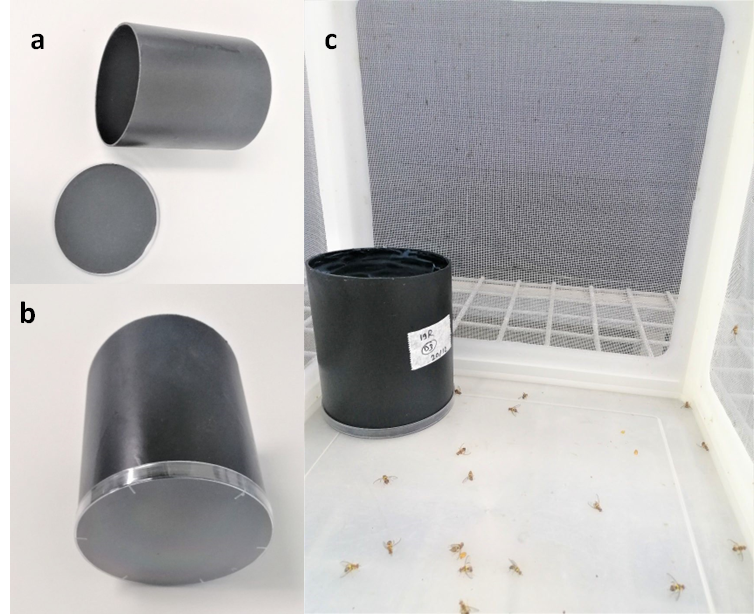


**Supplementary Fig. 2.** **a)** PVC cylinder lined with fluon on the inside, and Petri dish with a black cardboard; **b)** assembled flight ability test cylinder; **c)** dead and live Bactrocera tryoni adults that flew out of the cylinder (fliers) after EPN treatment.


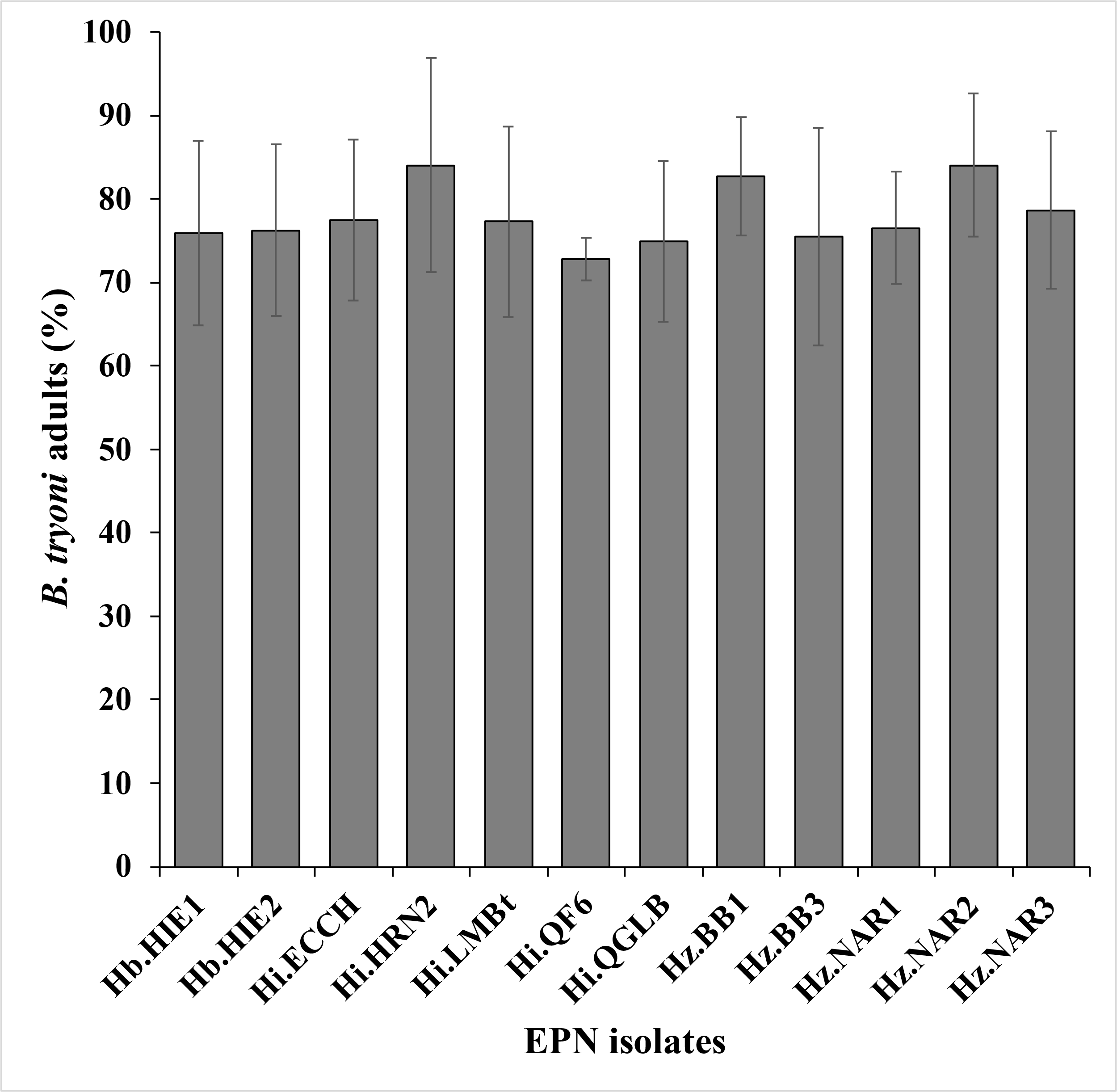


**Supplementary Fig. 3.** Corrected mortality of *Bactrocera tryoni* adults (%) after one week of feeding on an aqueous yeast hydrolysate (1% w/v) solution containing EPNs (500 IJs/mL), corrected by the mortality observed in the control treatment using Abbott’s formula (Abbott, 1925); hence no control mortality shown in this figure (but is shown in Fig. 1). Error bars indicate the standard deviation across five replicates. The EPN treatments were not significantly different from each other.
